# Acute reduction in posterior cerebral blood flow following isometric handgrip exercise is augmented by lower body negative pressure

**DOI:** 10.14814/phy2.13886

**Published:** 2018-10-18

**Authors:** Takuro Washio, Jennifer R. Vranish, Jasdeep Kaur, Benjamin E. Young, Keisho Katayama, Paul J. Fadel, Shigehiko Ogoh

**Affiliations:** ^1^ Department of Biomedical Engineering Toyo University Kawagoe‐shi Saitama Japan; ^2^ Research Fellow of Japan Society for the Promotion of Science Tokyo Japan; ^3^ Department of Kinesiology University of Texas at Arlington Arlington Texas; ^4^ Research Center of Health Physical Fitness and Sports Nagoya University Nagoya Japan

**Keywords:** Anterior cerebral blood flow, middle cerebral artery blood velocity, orthostatic stress, resistance exercise, syncope, vertebral artery blood flow

## Abstract

The mechanism(s) for the increased occurrence of a grayout or blackout, syncope, immediately after heavy resistance exercise are unclear. It is well‐known that orthostatic stress increases the occurrence of postexercise syncope. In addition, previous findings have suggested that hypo‐perfusion, especially in the posterior cerebral circulation rather than anterior cerebral circulation, may be associated with the occurrence of syncope. Herein, we hypothesized that the postexercise decrease in posterior, but not anterior, cerebral blood flow (CBF) would be greater during orthostatic stress. Nine healthy subjects performed 3‐min isometric handgrip (HG) at 30% maximum voluntary contraction without (CONTROL) and during lower body negative pressure (LBNP; −40 Torr) while vertebral artery (VA) blood flow, as an index of posterior CBF, and middle cerebral artery blood velocity (MCAv), as an index of anterior CBF, were measured. Immediately after HG (0 to 15 sec of recovery phase), mean arterial pressure decreased but there was no difference in this reduction between CONTROL and LBNP conditions (−15.4 ± 4.0% and −17.0 ± 6.2%, *P* = 0.42). Similarly, MCAv decreased following exercise and was unaffected by the application of LBNP (*P* = 0.22). In contrast, decreases in VA blood flow immediately following HG during LBNP were significantly greater compared to CONTROL condition (−24.2 ± 9.5% and ‐13.4 ± 6.6%, *P* = 0.005). These findings suggest that the decrease in posterior CBF immediately following exercise was augmented by LBNP, whereas anterior CBF appeared unaffected. Thus, the posterior cerebral circulation may be more sensitive to orthostatic stress during the postexercise period.

## Introduction

It is well‐known that there is an increased occurrence of a grayout or blackout, syncope, immediately after heavy physical work such as weight‐lifting (Compton et al. 1973). This phenomenon has been related to an inadequate cerebral perfusion and oxygenation (Van Lieshout et al. 2003; Franco 2007). In particular, posterior cerebral blood flow (CBF) regulation may be important because the posterior part of the cerebral circulation (the vertebral‐basilar system) supplies blood to the medulla oblongata, the location of cardiac, vasomotor, and respiratory control centers (Tatu et al. 1996). Indeed, recent studies have suggested that posterior cerebral hypo‐perfusion is involved in orthostatic syncope (Ogoh et al. 2015; Kay and Rickards 2016). Thus, we speculated that the response in the posterior cerebral circulation following resistance exercise may be a key factor regarding postexercise syncope. Importantly, previous studies investigating CBF regulation during and after resistance exercise have focused on anterior CBF (e.g., middle cerebral artery blood velocity, MCAv) and the response of posterior CBF remains unknown.

An emerging body of research has reported that posterior CBF regulation is different from that of the anterior cerebral circulation (Haubrich et al. 2004; Nakagawa et al. 2009; Sato et al. 2012a, 2012b; Ogoh et al. 2015). For example, orthostatic stress‐induced attenuation in dynamic cerebral autoregulation (CA) was much greater in the posterior cerebral circulation than anterior cerebral circulation (Sato et al. 2012a). This finding suggested that the response of posterior CBF to changes in perfusion pressure is augmented during orthostatic stress. Furthermore, it has been reported that there is an increased incidence of pre‐syncope following exercise during orthostatic stress (Eichna et al. 1947; Lacewell et al. 2014), indicating that orthostatic stress increases the risk of postexercise syncope (Krediet et al. 2004). However, whether the dynamics of posterior and anterior CBF regulation immediately following the cessation of exercise are affected differentially by a superimposed orthostatic stress remains unclear.

With this background in mind, we hypothesized that the post exercise decrease in posterior, but not anterior, CBF would be greater during an orthostatic stress. In order to examine the effect of an orthostatic stress on the regional CBF response immediately after exercise, we measured both vertebral artery (VA) blood flow as an index of posterior CBF and MCAv as an index of anterior CBF, before, during, and after isometric handgrip exercise without (CONTROL) and during lower body negative pressure (LBNP).

## Methods

### Subjects

Nine young men with a mean age of 25.0 ± 4.0 year, height of 177.5 ± 7.2 cm, and weight of 83.8 ± 5.3 kg, participated in this study. All subjects had no known cerebrovascular, cardiovascular, or pulmonary disorders and were not taking medications at the time of enrollment. In addition, subjects reported no history of pre‐syncope. Prior to each experimental session, participants were required to abstain from caffeinated beverages for 12 h, and strenuous exercise and alcohol for 24 h. Furthermore, the experiment was performed at least 3 h after a light meal. The protocol was approved by the Institutional Review Board at the University of Texas at Arlington (2016‐0783), and each subject provided written informed consent prior to participation in accordance with the principles of the Declaration of Helsinki.

### Experimental measurements

After arriving at the laboratory, subjects were positioned supine with their lower body in a LBNP chamber and sealed at the iliac crest. Participants were instrumented for measures of heart rate (HR) using a lead II electrocardiogram (Quinton Q710, Bothell, WA). Beat‐to‐beat arterial blood pressure (BP) was monitored continuously using finger photoplethysmography (Finapres Medical Systems, Amsterdam, the Netherlands) to determine systolic (SBP), diastolic (DBP), and mean arterial pressure (MAP). Return‐to‐flow calibrations were performed before each Finometer recording to ensure accurate measurements. Arterial BP was also measured periodically with an automated sphygmomanometer (Welch Allyn, Skaneateles Falls, NY) under resting conditions to further validate absolute BP values. Stroke volume (SV) and cardiac output (CO) were determined from the BP waveform using the Modelflow software program, which incorporates the sex, age, height, and weight of the subject (Beat Scope1.1; Finapres Medical Systems BV). End‐tidal carbon dioxide (EtCO_2_) was measured with a capnometer (Capnocheck Plus, Smith Medical, Dublin, OH). MCAv was measured through the left temporal window using a 2 MHz pulsed transcranial Doppler (TCD) probe (Multigon Industries*,* Inc., Yonkers, NY). The TCD probe was fixed and held in place using a headband. Right VA blood flow was measured between the transverse process of the C3 vertebra and subclavian artery using a color‐coded ultrasound system (Vivid i, GE Medical Systems) equipped with a 13.0 MHz linear transducer. When making blood velocity measurements, care was taken to ensure that the probe position was stable, that the insonation angle did not vary (60 deg in most cases), and that the sample volume was positioned in the center of the vessel and adjusted to cover the width of the vessel diameter but not extend beyond it (Thomas et al. 2015).

### Experimental protocol

Following instrumentation, each subject performed 3–5 maximal voluntary contractions (MVC) of handgrip (HG) on the right arm to determine the exercise intensity (30% MVC) for the experiment. In addition, subjects were briefly exposed to LBNP (30–60 sec) so they could hear the motor and feel the application of pressure on their lower body. Following that, the subjects rested in supine position on a bed for at least 10 min before the protocol commenced. Next, during a 3 min period while the subjects rested quietly, baseline measurements were made. Subjects then performed isometric HG for 3 min at 30% MVC. All cardiovascular measurements and MCAv measures were made continuously throughout, while VA blood flow measurements were made during the last minute of baseline and HG, and the first minute of recovery after the cessation of HG. During HG, the subjects were instructed to avoid performing a Valsalva maneuver and to also maintain the exercise intensity via visual feedback displayed on a computer screen. After the CONTROL trial, a minimum of 15 min rest period was provided to allow HR and BP to return to resting baseline values. Next, HG for 3 min at 30% MVC was repeated during the application of −40 Torr LBNP to simulate an orthostatic stress in the supine position (LBNP). Indeed, −40 Torr LBNP causes a similar orthostatic stress to standing up and assuming the upright position (Musgrave et al. 1969). For the LBNP trial, LBNP was turned on 1 min prior to the start of HG and was turned off after 1 min of recovery from HG. LBNP was applied throughout the protocol because applying it at the immediate offset of exercise would be a challenge to time appropriately, given the duration of time required for the chamber to reach −40 Torr. Subject stayed in the supine position throughout the protocol.

A HG and LBNP protocol was chosen to minimize movement and allow for accurate measurements of VA blood flow, which would be difficult during other forms of heavy exercise and moving the subject from the supine to upright position for an orthostatic challenge. Indeed, this protocol allowed for the subject's body position to be maintained relatively still during the performance of exercise with and without an orthostatic stress. The exercise intensity of 30% MVC performed for 3 min was chosen because it has been shown to elevate MAP by 20 to 30% (Linkis et al. 1995; Washio et al. 2017), which we reasoned would be sufficient to test our hypothesis while avoiding body movement and occurrences of Valsalva maneuvers compared with higher HG intensities or other forms of exercises (e.g., leg squats) (Perry et al. 2014).

### Data analyses

For Doppler calculations, the brightness mode was used to measure the systolic and diastolic diameters of the VA in a longitudinal section, and then the mean diameter was calculated: mean diameter (cm) = (systolic diameter)*1/3 +  (diastolic diameter)*2/3. Secondly, the pulse wave mode was used to measure the Doppler velocity spectrum, and ~10 to 20 cardiac cycles were averaged as a mean blood velocity (cm/seconds) to eliminate the effects caused by the breathing cycle (Ogoh et al. 2015; Thomas et al. 2015). Finally, blood flow was calculated by multiplying the mean cross‐sectional area with mean blood velocity; blood flow = [π*(mean diameter/2)^2^]*mean blood velocity*60 (mL/min). All blood flow measurements were performed by the same operator. MCA Cerebral vascular conductance index (MCA CVCi) or VA cerebral vascular conductance (VA CVC) was calculated from the ratio of MCAv or VA blood flow to MAP, respectively. Average values were calculated using 30 sec data points at the end of each condition (i.e., baseline, HG, and HG + LBNP). To identify the change in these parameters during recovery, 15 sec averages were calculated over the first minute of recovery: Re1, 0–15 sec average; Re2, 15–30 sec average; Re3, 30–45 sec average; Re4, 45–60 sec average. The TCD signal in one subject was lost during exercise, thus TCD data are presented for eight subjects.

### Statistical analysis

All values are expressed as mean ± SD. Normal distribution was tested using Shapiro‐Wilk Tests. Two‐way analysis of variance (ANOVA) with repeated measures (condition × time) was performed with Bonferroni's post hoc test (SPSS 24, IBM, Tokyo, Japan). Pearson correlation was used to analyze the statistical relationship between relative changes in VA blood flow or MCAv and MAP after HG (0–15 sec after HG) without (CONTROL) and during LBNP. A *P*‐value less than 0.05 was regarded as significant.

## Results

There was a significant main effect of LBNP on HR, SBP, MAP, SV, CO, and EtCO_2_. In addition, there was a significant time effect on HR, SBP, DBP, MAP, CO, and EtCO_2_, however no significant interactions were present for any of these variables (Table 1).

**Table 1:** Cardiovascular measurements at resting baseline, during handgrip exercise and recovery

	Baseline	HG	Re1	Re2	Re3	Re4	*P* value		
							LBNP	Time	Interaction
HR (beats/min)									
CONTROL	60.6 ± 12.2	83.7 ± 13.0	72.3 ± 13.8	66.3 ± 12.4	61.0 ± 11.9	60.5 ± 11.4	0.004	<0.001	0.823
LBNP	71.5 ± 14.2	95.4 ± 19.2	85.2 ± 21.4	76.2 ± 19.9	73.8 ± 16.2	74.0 ± 16.4			
SBP (mmHg)									
CONTROL	133.8 ± 14.1	166.0 ± 16.6	148.1 ± 17.0	139.3 ± 16.4	140.4 ± 14.8	138.8 ± 13.8	0.023	<0.001	0.160
LBNP	130.2 ± 21.9	163.2 ± 23.5	142.6 ± 21.2	127.9 ± 17.4	126.9 ± 15.4	125.9 ± 9.7			
DBP (mmHg)									
CONTROL	71.1 ± 8.1	91.9 ± 8.0	74.9 ± 8.7	72.8 ± 7.5	74.8 ± 7.1	75.4 ± 6.2	0.828	<0.001	0.552
LBNP	71.6 ± 10.6	93.8 ± 11.5	74.5 ± 10.6	71.6 ± 8.7	74.5 ± 7.0	73.6 ± 4.8			
MAP (mmHg)									
CONTROL	91.8 ± 10.7	120.2 ± 10.5	101.8 ± 11.6	95.9 ± 10.0	97.7 ± 9.0	97.4 ± 8.1	0.003	<0.001	0.747
LBNP	88.3 ± 12.8	115.6 ± 10.7	95.8 ± 10.8	89.3 ± 9.4	91.6 ± 7.6	90.6 ± 4.0			
SV (mL)									
CONTROL	116.6 ± 13.3	122.6 ± 12.3	126.5 ± 11.3	125.8 ± 11.4	125.4 ± 9.2	121.8 ± 8.5	<0.001	0.157	0.211
LBNP	90.8 ± 14.0	92.3 ± 18.6	97.7 ± 13.3	94.4 ± 10.7	88.3 ± 13.2	88.5 ± 12.9			
CO (L/min)									
CONTROL	7.1 ± 1.9	9.6 ± 1.9	9.2 ± 2.3	8.4 ± 2.0	7.7 ± 1.6	7.4 ± 1.5	0.003	0.002	0.639
LBNP	6.5 ± 1.6	8.4 ± 1.5	8.2 ± 1.8	7.1 ± 1.5	6.4 ± 1.2	6.5 ± 1.4			
EtCO_2_ (mmHg)									
CONTROL	42.6 ± 2.8	41.4 ± 3.3	41.0 ± 2.2	42.5 ± 2.0	42.1 ± 2.2	40.4 ± 3.2	<0.001	0.012	0.505
LBNP	40.2 ± 3.1	39.3 ± 3.4	38.0 ± 2.2	39.5 ± 1.7	39.8 ± 2.4	39.3 ± 2.4			

Data are mean ± SD (*n* = 9); HG, handgrip exercise; Re1, postexercise 0–15 sec; Re2, postexercise 15–30 sec; Re3, postexercise 30–45 sec; Re4, postexercise 45–60 sec HR, heart rate; SBP, systolic blood pressure; DBP, diastolic blood pressure; MAP, mean arterial pressure; SV, stroke volume; CO, cardiac output; EtCO_2_, End‐tidal carbon dioxide.

VA blood flow increased significantly during HG and returned to the baseline values after the exercise (*P* < 0.001). LBNP did not change VA blood flow response during the exercise protocol (*P* = 0.355), however, VA blood flow was lower at Re1 (0–15 sec after HG) during LBNP compared with the CONTROL condition (117.5 ± 30.5 vs. 133.7 ± 27.2 mL/min, *P* = 0.048). MCAv responses to HG and recovery from HG were similar between LBNP and CONTROL (*P* = 0.421, Table 2). There was no significant LBNP × time interaction for MCAv (Table 2).

**Table 2:** Cerebral vascular measurements at resting baseline, during handgrip exercise and recovery

	Baseline	HG	Re1	Re2	Re3	Re4	*P* value		
							LBNP	Time	Interaction
VA blood flow (mL/min)									
CONTROL	118.4 ± 24.0	153.7 ± 25.7†	133.7 ± 27.2† ^,^ §	122.3 ± 24.1§	122.1 ± 21.2§	115.2 ± 23.2§	0.355	<0.001	0.014
LBNP	115.1 ± 24.9	153.3 ± 26.2†	117.5 ± 30.5* ^,^ §	119.2 ± 24.8§	116.0 ± 24.2§	116.8 ± 23.2§			
VA CVC (mL/min/mmHg)									
CONTROL	1.31 ± 0.34	1.28 ± 0.23	1.32 ± 0.28	1.30 ± 0.35	1.26 ± 0.27	1.19 ± 0.28	0.622	0.354	0.040
LBNP	1.33 ± 0.37	1.33 ± 0.23	1.22 ± 0.25	1.35 ± 0.33	1.27 ± 0.28	1.29 ± 0.29			
MCAv (cm/s) *n* = 8									
CONTROL	58.1 ± 10.8	67.0 ± 12.2	64.1 ± 9.7	60.7 ± 10.0	59.6 ± 9.6	59.3 ± 11.9	0.421	<0.001	0.247
LBNP	59.1 ± 11.8	68.1 ± 11.8	62.6 ± 9.1	58.1 ± 8.8	59.0 ± 9.9	56.4 ± 9.9			
MCA CVCi (cm/s/mmHg) *n* = 8									
CONTROL	1.64 ± 0.41	1.84 ± 0.37	1.61 ± 0.33	1.62 ± 0.32	1.68 ± 0.31	1.72 ± 0.36	0.016	0.001	0.837
LBNP	1.54 ± 0.38	1.73 ± 0.38	1.53 ± 0.25	1.55 ± 0.22	1.57 ± 0.23	1.64 ± 0.27			

Data are mean ± SD (*n* = 9); HG, handgrip exercise; Re1, postexercise 0–15 sec; Re2, postexercise 15–30 sec; Re3, postexercise 30–45 sec; Re4, postexercise 45–60 sec VA CVC, vertbral artery cerebral vascular conductance; MCA CVCi, middle cerebral artery cerebral vascular conductance index. *P < 0.05, versus CONTROL. †*P* < 0.05, versus baseline. §*P* < 0.05, versus HG. One subject was excluded from the analysis due to excessive noise in the MCAv signal.

**Figure 1:**
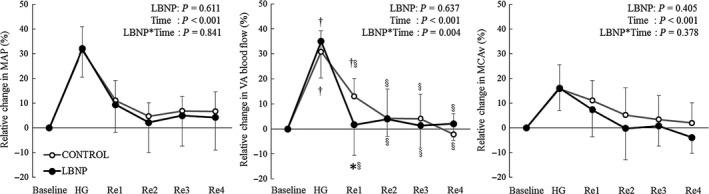
Relative changes in mean arterial pressure (MAP), vertebral artery (VA) blood flow, and middle cerebral artery velocity (MCAv, *n* = 8) from each resting baseline. Data are mean ± SD (*n* = 9); HG, handgrip exercise; Re1, post exercise 0–15 sec; Re2, postexercise 15–30 sec; Re3, postexercise 30–45 sec; Re4, postexercise 45–60 sec. *P < 0.05, versus CONTROL. ^†^ *P* < 0.05, versus baseline. ^§^ *P* < 0.05, versus HG.

**Figure 2:**
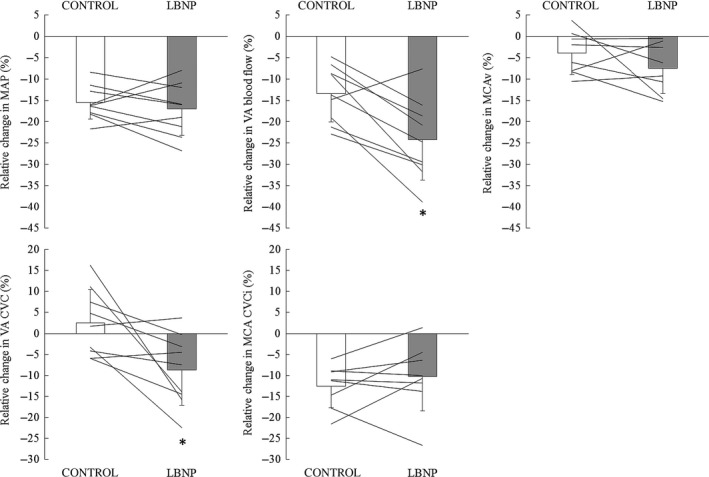
Relative changes in mean arterial pressure (MAP), vertebral artery (VA) blood flow, middle cerebral artery velocity (MCAv, *n* = 8), VA cerebral vascular conductance (VA CVC), and MCA cerebral vascular conductance index (MCA CVCi) from handgrip (HG) to postexercise recovery (Re1, 0–15 sec) with or without lower body negative pressure. Data are mean ± SD (*n* = 9). *P < 0.05, versus CONTROL.

**Figure 3:**
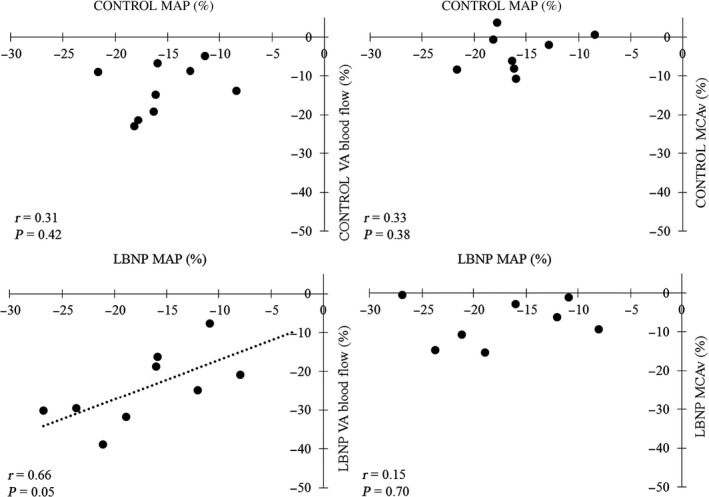
Relationship between relative changes in vertebral artery (VA,* n* = 9) blood flow or middle cerebral artery velocity (MCAv, *n* = 8) and mean arterial pressure (MAP) from handgrip to postexercise recovery (Re1, 0–15 sec) with or without lower body negative pressure.

The application of LBNP modified the percent change in VA blood flow across the protocol (Fig. 1, *P* = 0.004) despite no effect of LBNP on the changes in MAP and MCAv (*P* = 0.841 and *P* = 0.378, respectively). In particular, the reduction in VA blood flow during Re1 (0–15 sec after HG) was greater during LBNP (−24.2 ± 9.5%, Fig. 2) compared with that during CONTROL condition (−13.4 ± 6.6%, *P* = 0.005) despite no change in MAP (*P* = 0.416) and MCAv (*P* = 0.217). Similarly, there was a significant reduction in the relative change in VA CVC from HG to recovery 1 (0–15 sec after HG) between LBNP and the CONTROL condition (−8.7 ± 8.5% vs. 2.5 ± 8.0%, *P* = 0.022) but not in MCA CVCi (*P* = 0.390). In addition, relative changes in VA blood flow at Re1 (0‐15 sec after HG exercise) correlated with relative changes in MAP during LBNP (*r* = 0.66, *P* = 0.05) but not without LBNP (CONTROL: *r* = 0.15, *P* = 0.70). In contrast, relative changes in MCAv did not correlate with changes in MAP regardless of the application of LBNP (Fig. 3).

## Discussion

The present study for the first time assessed the effect of orthostatic stress on both anterior and posterior CBF responses during and immediately after exercise. The major novel finding was that acute reductions in posterior CBF following isometric exercise were augmented during LBNP, whereas anterior CBF appeared unaffected. These findings suggest that the posterior cerebral circulation may be more sensitive to orthostatic stress during the post exercise period as compared with the anterior cerebral circulation.

Postexercise syncope can be defined as a loss of consciousness or development of pre syncopal signs and symptoms during recovery from a bout of physical activity or exercise (Halliwill et al. 2014). Regarding this phenomenon, many previous studies have investigated the physiological responses to post‐prolonged dynamic exercise since this exercise condition evokes reductions in BP as postexercise hypotension that may overlap with postexercise syncope (Noakes 1988; Holtzhausen et al. 1994; Holtzhausen and Noakes 1995, 1997; Kenefick and Sawka 2007). Notably, the physiological response to resistance exercise is different from that of dynamic exercise (Romero et al. 2017). For example, post resistance exercise syncope can occur during the exercise or within seconds of completion (Compton et al. 1973). Importantly, compared with dynamic exercise, arterial BP increases profoundly during resistance exercise and subsequently it falls rapidly to pre exercise values or below upon the completion of the exercise bout (MacDougall et al. 1985; Edwards et al. 2002; Pott et al. 2003; Romero and Cooke 2007). Consequently, cerebral perfusion falls rapidly immediately following resistance exercise (Edwards et al. 2002; Romero and Cooke 2007). This reduction may be associated with the large and rapid decrease in BP. Therefore, the precipitous acute drop in BP following resistance exercise may be a sufficient challenge to the cerebral circulation to evoke syncope (MacDougall et al. 1985; Dickerman et al. 2000). In addition, previous studies suggest that orthostatic stress (Mundel et al. 2015) and resistance exercise‐induced hyperventilation (Compton et al. 1973; Romero and Cooke 2007) affect CBF and may contribute to post resistance exercise syncope. Interestingly, the rapid fall in MCAv following dynamic resistance exercise does not appear to cause dizziness or syncope (Edwards et al. 2002). Also, orthostatic stress‐induced decreases in anterior CBF are not associated with orthostatic intolerance (Ogoh et al. 2015). Taken together, these findings suggest that reductions in anterior CBF may not contribute importantly to syncopal events.

It is noteworthy that all of the previous studies regarding post exercise syncope have focused on the blood flow response in the anterior cerebral circulation. Since the posterior cerebral circulation supplies blood to the medulla oblongata, the location of important cardiac, vasomotor, and respiratory control centers (Tatu et al. 1996), it is possible that the posterior cerebral circulation is more important during orthostatic stress. Indeed, cerebral hypo‐perfusion of the posterior regions of the brain may be associated with orthostatic intolerance (Ogoh et al. 2015; Kay and Rickards 2016). Thus, in the present study, we expected that decreases in posterior CBF rather than anterior CBF would be an important key factor associated with post resistance exercise syncope and we for the first time examined changes in posterior CBF following resistance exercise with and without an orthostatic stress.

Herein, we demonstrated that although the response of anterior CBF (MCAv) post exercise was not altered by the application of LBNP, the reduction in posterior CBF (VA blood flow) was augmented by LBNP (Fig. 2). It is well‐known that there is an increased incidence of pre syncope during orthostatic stress following exercise (Eichna et al. 1947; Lacewell et al. 2014). Moreover, standing or head‐up tilt can lead to cardiovascular collapse in the form of pre syncopal signs and symptoms, or even frank syncope if orthostatic stress is maintained (Blomqvist and Stone 1983). On the other hand, visual‐cognitive deficits occur during post exercise pre syncope (Sieck et al. 2016). The function of visual cortex is associated with posterior CBF rather than anterior CBF (Aaslid 1987) and severe orthostatic stress can evoke a large reduction in posterior CBF (Ogoh et al. 2015). Therefore, based on these previous works and the findings of the present study, it is reasonable to suggest that the posterior CBF response after exercise was augmented by orthostatic stress and this phenomenon may be one key physiological factor associated with syncope following resistance exercise. Nevertheless, we did not identify pre syncope or syncope in the present study. Therefore, further investigations are needed to determine the effect of this augmented decrease in posterior CBF during orthostatic stress following isometric exercise on post exercise pre syncope or syncope.

The underlying mechanisms for different blood flow responses between the anterior and posterior cerebral circulation remain unknown; however, some possibilities warrant discussion. One possible explanation is different dynamic CA between the anterior and posterior cerebral circulations (Sato et al. 2012a). In this regard, our previous study (Sato et al. 2012a) demonstrated that orthostatic stress induced an impairment in dynamic CA in the posterior cerebral circulation that was greater compared with that in the anterior cerebral circulation. In the present study, the reduction in VA blood flow immediately after exercise during LBNP was related to that of MAP but this statistical relationship was not observed for the anterior cerebral circulation (Fig. 3). These findings are supported by previous work reporting that orthostatic stress‐induced attenuation in dynamic CA at posterior cerebral circulation is larger than that at anterior circulation (Sato et al. 2012a). In addition, other factors such as sympathetic innervation of cerebral arteries and cerebrovascular CO_2_ reactivity may be involved. The posterior cerebral circulation may have less sympathetic innervation than the anterior cerebral portion (Edvinsson et al. 1976). In addition, posterior cerebral circulation has lower CO_2_ reactivity than the anterior cerebral circulation (Sato et al. 2012b). The potential influence of these different physiological factors between anterior and posterior cerebral circulation on the response of CBF at each artery following exercise requires further study.

In the present study, we used small muscle mass exercise (HG) with a moderate intensity, which alone does not cause post exercise syncope or large acute hypotension. However, this level of HG increased BP approximately 30% allowing for a significant pressor response and a rapid reduction in BP following the cessation of exercise. Thus, although we could not assess the relationship between onset of syncope or pre syncope and CBF responses, we were able to present a significant challenge to the cerebral circulation. Importantly, with the application of LBNP, we were also able to apply and control a consistent orthostatic stress to further challenge the cerebral vasculature, while being able to quantify VA blood flow. Indeed, we were limited by the technical issues associated with movement when making duplex Doppler ultrasound measurements. Nevertheless, we provide novel insight into the effect of orthostatic stress on both anterior and posterior CBF responses immediately after exercise that may be a key physiological factor associated with post exercise syncope. Notably, since higher workloads or different modes of exercise would cause greater BP responses and also, may induce syncope, we suspect that differential responses between the anterior and posterior cerebral circulations would be even more robust; however, additional studies are needed. Also, since severe resistance exercise often causes spontaneous Valsalva maneuvers and, Compton et al. (1973) pointed out that weight‐lifters’ blackout can be attributed to the reduced CBF‐associated with the Valsalva maneuver, this aspect of resistance exercise training warrants future investigation. In the present study, subjects were asked to avoid performing a Valsalva maneuver or holding their breath as the exercise became more difficult, thus, our findings are independent of any influence of a Valsalva maneuver.

The increase in CBF during exercise is detected with a parallel increase in brain neuronal activity and metabolism (Ogoh and Ainslie 2009). In addition, right handed contraction provokes an elevation in left MCAv (contralateral side) that is associated with cerebral neural activation in the motor‐sensory cortex and supplementary motor area (Linkis et al. 1995). In the present study, we measured cerebral blood velocity at the left side of MCA during right handed exercise, suggesting that the results regarding MCAv during exercise was reflected by changes in cerebral neural activity. In other words, we cannot rule out the possibility that the response of right MCAv (ipsilateral side) may cause different results compared with contralateral side. Similarly, in the present study, we measured VA blood flow on the right, ipsilateral side of HG. Nevertheless, our previous study demonstrated no significant difference between right (ipsilateral side) and left (contralateral side) VA blood flow during HG (Washio et al. 2017). This result is reasonable because the basilar system integrates both right and left VA blood flow and VA does not supply blood to motor‐sensory cortex and supplementary motor area. Thus, taken together, the measurement of VA blood flow on the left, contralateral side, should not modify our conclusions.

Some potential limitations of the present study should be considered. We did not use duplex Doppler ultrasound to measure anterior CBF because only one ultrasound unit was available at the time of the study. However, the TCD‐determined cerebral blood velocity can be used as an index of CBF with the assumption of constant diameter of the insonated artery. In this regard, several previous studies have demonstrated no change in MCA diameter in response to orthostatic stress (Serrador et al. 2000) or changes in arterial carbon dioxide (Giller et al. 1993; Coverdale et al. 2014). Although more recent reports demonstrated that 60% MVC rhythmic HG (Verbree et al. 2017) or severe hypercapnia (Verbree et al. 2014) caused significant changes in MCA diameter, these conditions were much stronger compared to our experimental protocol. In the present study, throughout the protocol, changes in EtCO_2_ were small and sympatho‐excitation and fatigue likely lower than 3 consecutive 5‐min bouts of 60% MVC rhythmic HG exercise. A second limitation to consider is that we used small muscle mass HG as our mode of resistance exercise, which may not be reflective of larger muscle mass resistance exercise (e.g., leg press). This was necessitated because of the sensitivity to movement of the measurements made and for the application of LBNP, in particular immediately following exercise. Although MAP decreased rapidly following HG exercise this reduction is not consistent with previous studies using leg press (Edwards et al. 2002; Moralez et al. 2012) that showed decreases in MAP to below baseline values (i.e., post exercise hypotension). Nevertheless, importantly, the reduction in MAP was rapid and not different between CONTROL and LBNP conditions. Third, we only recruited male subjects in the present study and our findings should not be extrapolated to women until future studies are performed. This is important because previous research has indicated that there is an increased cerebral vascular resistance at the MCA in the luteal compared to the follicular phase of the menstrual cycle (Brackley et al. 1999). However, the effect of estrogen on cerebral vasculature is controversial (Gros et al. 2002) and no study has examined the effect of gender on posterior CBF. Finally, it has been shown previously that cerebrovascular responses differ between LBNP and head up tilt despite inducing similar systemic hemodynamic responses (Bronzwaer et al. 2017). Thus, CBF responses can be affected by hydrostatic pressure gradient, and this impact on post exercise syncope and the response of regional CBF to orthostatic stress following exercise requires further studies.

In summary, the application of LBNP augmented the reduction of posterior CBF following resistance exercise, whereas anterior CBF appeared unaffected. These findings suggest that the posterior cerebral circulation is more sensitive to orthostatic stress during the post exercise period as compared with the anterior cerebral circulation.

## Conflict of Interest

No conflicts of interest, financial or otherwise, are declared by the author(s).
